# Intraoperative ultrasound-guided iodine-125 seed implantation for unresectable pancreatic carcinoma

**DOI:** 10.1186/1756-9966-28-88

**Published:** 2009-06-23

**Authors:** Junjie Wang, Yuliang Jiang, Jinna Li, Suqing Tian, Weiqiang Ran, Dianrong Xiu

**Affiliations:** 1 Department of Radiation Oncology, Peking University 3rd Hospital, Beijing, 100083, PR China; 2 Department of Ultrasound, Peking University 3rd Hospital, Beijing, 100083, PR China; 3 Department of Surgery, Peking University 3rd Hospital, Beijing, 100083, PR China

## Abstract

**Background:**

To assess the feasibility and efficacy of using ^125^I seed implantation under intraoperative ultrasound guidance for unresectable pancreatic carcinoma.

**Methods:**

Fourteen patients with pancreatic carcinoma that underwent laparotomy and considered unresectable were included in this study. Nine patients were pathologically diagnosed with Stage II disease, five patients with Stage III disease. Fourteen patients were treated with ^125^I seed implantation guided by intraoperative ultrasound and received D_90 _of ^125^I seeds ranging from 60 to 140 Gy with a median of 120 Gy. Five patients received an additional 35–50 Gy from external beam radiotherapy after seed implantation and six patients received 2–6 cycles of chemotherapy.

**Results:**

87.5% (7/8) of patients received partial to complete pain relief. The response rate of tumor was 78.6%, One-, two-and three-year survival rates were 33.9% and 16.9%, 7.8%, with local control of disease achieved in 78.6% (11/14), and the median survival was 10 months (95% CI: 7.7–12.3).

**Conclusion:**

There were no deaths related to ^125^I seed implant. In this preliminary investigation, ^125^I seed implant provided excellent palliation of pain relief, local control and prolong the survival of patients with stage II and III disease to some extent.

## Introduction

The incidence of pancreatic carcinoma has increased in recent decades, yet the treatment outcome for this disease remains unsatisfactory. Despite the introduction of new therapeutic techniques combined with aggressive modalities, such as external beam radiotherapy (EBRT) and chemotherapy, the prognosis of pancreatic carcinoma remained to be very poor, with a mortality rate of more than 90% [1]. Only 15% to 20% of patients with pancreatic carcinoma are suitable for resection, and even with resection, long term survival still remains poor [2,3]. Most of pancreatic carcinoma was diagnosed in the locally advanced or metastatic stage, and the median survival rate was approximately 6 months with palliative treatment. Biliary and gastric bypass have been used for palliation in unresectable pancreatic carcinomas and median survival in these patients was often 5–6 months [4,5].

More recently, EBRT and chemotherapy have been standard adjuvants for locally advanced pancreatic carcinoma. EBRT alone has failed to control disease progression and yields a median survival of 5.5–7 months [6,7], while the addition of chemotherapy to EBRT increased the median survival to 9–10 months [8-10]. The introduction of intraoperative electron beam radiotherapy, combined with EBRT and chemotherapy, has also failed to significantly improve long-term results, with recent studies reporting median survival rates of 7–16 months [11-14].

Despite the availability of many treatments, there was currently no consensus regarding the optimal therapeutic modality for unresectable pancreatic carcinomas. Therefore, it is necessary to investigate new techniques that may improve the prognosis. In this study we investigated the efficacy and feasibility of ^125^I seed implantation guided by intraoperative ultrasound in managing unresectable pancreatic carcinoma.

## Methods

### Patient information and selection

Between October 2003 and February 2006, 14 patients with a Karrnofsky performance status (KPS) score of 70 or above (which is associated with a survival of >3 months) were identified. Of these 14 patients, 50% (7/14) demonstrated jaundice, 57% (8/14) suffered from pain, 21% (3/14) suffered from intestinal obstruction and 93% (13/14) experienced weight loss. These patients were evaluated as unresectable pancreatic carcinoma by surgeons during laparotomy and received ^125^I seed implantation guided by intraoperative ultrasound. The criteria of unresectable diseases included vascular invasion or vascular invasive combined with metastasis to the local region lymph nodes. Of the 14 pancreatic carcinoma patients, 9 were diagnosed with stage II disease, 5 patients with stage III disease. A summary of patient characteristics is listed in Table 1, Table 2 and Additional file 1. Two of the patients with jaundice did receive a biliary stent treatment one month before ^125^I seed implantation. All patients were evaluated for the extent of disease progression by physical examination, complete blood panel, chest X-ray, abdominal CT scans and ultrasound before seed implantation. This study was approved by the institutional review board and informed consent was obtained.

**Table 1:** Summary of patient characteristics (n = 14)

	**No of patients**	**%**
Gender		
Male	7	50
Female	7	50
Stage II	9	64
pT3N0M0	6	
pT1N1M0	0	
pT2N1M0	0	
pT3N1M0	3	
Stage III	5	36
pT4N any Mo	5	
Primary tumor location		
Head	6	**44**
Body and/or tail	3	21
Head and body/tail	3	21
Whole pancreas	2	14
Symptoms		
Jaundice	7	50
Pain	8	57
Weight loss	13	93
Intestinal obstruction	3	21
Pathology		
Adenocarcinoma	14	100

**Table 2:** Characteristics of patients and treatment (14)

No	Gender	Age	TNM	Stage	KPS	Jaundice	Surgery	Other treatment	Adjunant EBRT	Adjunant CTx (cycles)
1	F	64	pT3N0M0	II	80	Yes	biliary enteric anastomosis +gastrojejunostomy	No	35 Gy/17 f	PTX(2)
2	M	53	pT3N0M0	II	80	Yes	No	PTCD+stent	No	No
3	F	71	pT3N0M0	II	80	No	No	PTCD+stent	No	No
4	F	66	pT3N1M0	II	80	No	No	No	No	No
5	M	65	pT3N0M0	II	80	Yes	biliary enteric anastomosis	No	No	No
6	M	75	pT3N0M0	II	70	Yes	biliary enteric anastomosis +gastrojejunostomy	No	No	No
7	F	48	pT4N0M0	III	80	Yes	biliary enteric anastomosis +gastrojejunostomy	No	40 Gy/20 f	PTX(2) GEM(2)
8	M	62	pT3N1M0	II	70	Yes	biliary enteric anastomosis	No	No	No
9	F	38	pT3N1M0	II	70	Yes	biliary enteric anastomosis	PTCD+stent	No	No
10	M	46	pT4N0M0	III	70	No	No	No	40 Gy/20 f	GEM(4)
11	F	56	pT4N0M0	III	80	No	No	No	No	GEM(3)
12	M	41	pT4N0M0	III	80	No	No	No	No	No
13	F	43	pT3N0M0	II	90	No	No	No	50 Gy/25 f	GEM(6)
14	M	52	pT4N1M0	III	80	No	No	No	50 Gy/25 f	No

Abbreviations: KPS: Karnofsky; EBRT: external beam radiotherapy; CTx: chemotherapy; PTBD: percutaneous transhepatic biliary drainage; PTX: paclitaxel; Gem: gemcitabine.

### Treatment planning

Patients underwent a detailed tumor volume study using CT scans 1–2 weeks before seed implantation. Images of each pancreatic carcinoma were obtained at 5 mm intervals. The radiation oncologist and surgeons together outlined the gross tumor volume (GTV) on each image and planning target volume (PTV) included GTV plus 0.5 – 1.0 cm peripheral tissue. These tracings were digitized and scanned to define the tumor volume, from which the D_90 _of 60–140 Gy for ^125^I seed irradiation, with the median of 120 Gy and the number of ^125^I seeds to be implanted could be calculated. The D_90 _was prescribed in a way that at least 90% of the tumor volume received the reference dose. The ^125^I seeds used (Beijing Atom and High Technique Industries Inc, Beijing, Modle-6711) had a half-life t_1/2 _of 59.4 days with a low energy level of 27.4 KeV and a half-value of 0.025 mm in lead. The computer treatment planning system (Beijing Fei Tian Technique Industries Inc, Beijing, China) was used for dose calculations.

### Treatment technique

**Figure 1:**
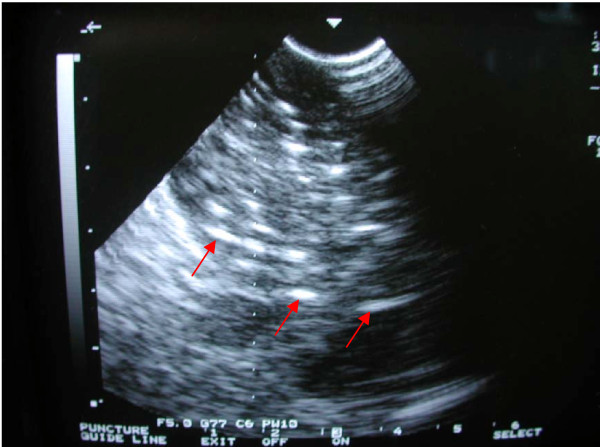
**Intraoperative ultrasound scan showing the distribution of implanted seeds in the tumor**.

After the diagnosis of pancreatic cancer had been established by biopsy intraoperation, tumor volume was measured during laparotomy by intraoperative ultrasonography utilizing a megahertz linear probe. Guided by ultrasound, 18-gauge needles were implanted into mass and spaced in a parallel array at intervals of 1.0 cm, extending at least 0.5~1 cm beyond the margins of the pancreatic lesions. During the placement of the needles, care was taken to avoid the needles from the pancreatic duct, small blood vessels, and the adjacent transverse colon at least 1 cm. After needles were implanted, ^125^I seeds were implanted using a Mick-applicator and the spacing was maintained at 1.0 cm intervals (Figure 1). The number of ^125^I seeds implanted ranged from 10 to 75, with the median number implanted of 38. The specific activity of ^125^I ranged from 0.40 to 0.60 mCi per seed, and the total isotope radioactivity implanted ranged from 4 to 37.5 mCi. An omental fat pad was placed over the implanted volume to protect the gastric and transverse colon mucosa from irradiation. Postimplant EBRT was generally recommended to all patients for an adjuvant aim, but only 5 patients received EBRT at 4–6 weeks after ^125^I seed implantation. The total doses of EBRT ranged from 35 to 50 Gy at 1.8–2.0 Gy per fraction. Postoperative chemotherapy was recommended to all patients on an adjuvant or palliative basis, but only six patients received chemotherapy consisted of Gemcitabine or Paclitaxel (PTX) and was completed 2 to 6 cycles. The other patients refused to receive EBRT or chemotherapy furthermore after seed implantation.

### Definition for the clinical benefit response

The pain intensity was evaluated and graded by the International Association for the Study of Pain [15]. Numerical Rating Scale (NRS) 1–3 of pain was mild, NRS 4–6 was moderate and NRS 7–10 was severe. The complete response (CR) was no pain after seed implant, partial response (PR) was pain relief, pain-free sleep and maintenance of a normal life. No response (NR) was meaning no change of pain severity compared with pre-seed implant. The response rates (RR) of pain relief were defined as moderate and severe pain decreasing to mild pain; the RR was CR + PR. Tumor responses and toxicity were assessed using WHO criteria [16]. In brief, a complete response (CR) was defined as the complete disappearance of all measurable lesions, without the appearance of any new lesion. A partial response (PR) was defined as a reduction in bidimensionally measurable lesions by at least 50 percent of the sum of the products of their largest perpendicular diameters and an absence of progression in other lesions, without the appearance of any new lesion. Stable disease (SD) was defined as a reduction in tumor volume of less than 50 percent or an increase in the volume of one or more measureable lesions of less than 25 percent, without the appearance of any new lesion. Progressive disease (PD) was defined as an increase in the size of at least 25% percent and the appearance of any new lesions. The response rate was CR + PR.

### Follow-up and statistical analyses

One month after seed implantation, patients were evaluated by radiation oncologists and surgeons by physical examination, complete blood panel, chest X-ray, abdominal CT and ultrasound. One month later, a clinical consultation was provided. After that, evaluation was given every 2–3 months or sooner if a new clinical sign or symptom appeared. Time of survival was calculated from the date of diagnosis to the date of death or last follow-up. A local recurrence was defined as tumor progression (PD) within the implanted area or surrounding regions as seen on CT. Local recurrence and distant metastasis were scored until patient death and censored thereafter. Overall survival curves were generated using the Kaplan-Meier method using SPSS10.0 and deaths for any reason were scored as events.

## Results

### Relief of pain symptoms

Pain was the presenting symptom in 57.1% (8/14) of patients prior to treatment. Following ^125^I seed implantation, the RR was 87.5% (7/8), two of patients with severe pain become no pain, two of patients with severe pain become mild pain, one of patients with severe pain became moderate, two of patients with moderate pain became no pain and one of patients with moderate became mild pain. Most patients experienced pain relief within one week following seed implantation.

### Local control and survival

**Figure 2:**
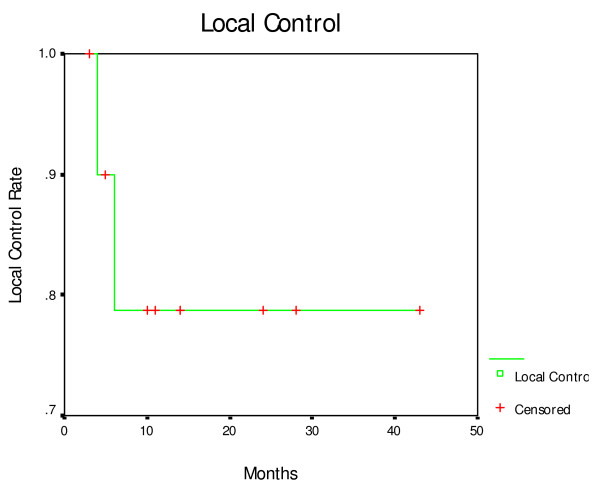
**Actuarial local control curve for 14 patients treated with ^125 ^I seed implantation**.

**Figure 3:**
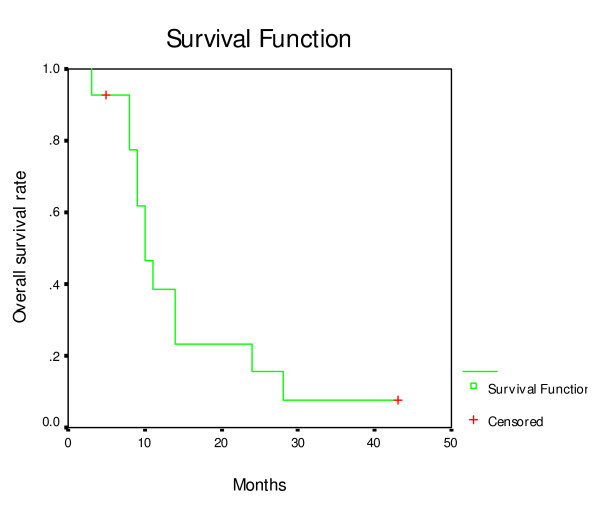
**Actuarial survival curve for 14 patients with unresected stage II/III pancreatic carcinoma treated with ^125^I seed implantation**.

The response rate of tumor was 78.6%, overall local control rates in this study were 78.6% (11/14) (Figure 2) too. The overall median survival was 10 months (95% CI, 7.6–12.3), while the overall 1-, 2- and 3-year survival rates were 33.9%, 16.9% and 7.8%, respectively. The Kaplan-Meier actuarial survival curve of all 14 patients treated with seed implantation is shown in Figure 3. Seven patients died of metastases to the liver and peritoneal surface, yet had no image evidence of any residual local disease. Two patients died of local progression, two patients died of local progression and metastases, one patient died of heart disease.

### Toxicity and complications

No patient died during the perioperative period, although chylous fistula was observed in one patient (7%). One patient (7%) who underwent both seed implantation and EBRT developed a gastric ulcer. One patient (7%) experienced radiation enteritis and 7 (50%) patients experienced fever. Clinical evaluation, ultrasound, and CT scans determined that the majority of patients developed metastases to the liver and peritoneal surface. Additionally, for 2 (14%) patients, three seeds were found to have migrated to the liver in each case. However, no side effects were observed for 12-months post-treatment.

## Discussion

The treatment of unresectable pancreatic cancer continues to be a major challenge. More than half of patients have a locally or regionally confined tumor requiring local treatment. Stereotactic radiotherapy (SRT) allows an escalation of radiation doses to be applied to a small target volume within a small margin. SRT is administered in one or a few fractions with the goal of sparing the surrounding normal tissue by using multiple non-coplanar field arrangements for the administration. In a phase II study on the use of SRT in the treatment of locally advanced pancreatic carcinoma by Huyer et al, the median survival time was only 5.7 months, and the one-year survival rate was 5% [17]. These data associate SRT with a poor outcome, unacceptable toxicity, and questionable palliative effects, making SRT unadvisable for patients with advanced pancreatic carcinoma. In contrast, interstitial permanent implantation of radioactive seeds into the tumor site provides the advantage of delivering a high dose of irradiation to the tumor (range 140–160 Gy) which drops off sharply outside the local implanted field. ^125^I seeds with a half-life of approximately 59.4 days were selected as the radioactive source for permanent implantation in this study, allowing approximately 95% of the needed dose to be delivered within a year [18].

Implantation of radioactive isotopes for the treatment of pancreatic carcinoma has been used for the past several decades. For example, Handly et al. reported the use of radium needle implantation in 7 patients for the treatment of pancreatic carcinoma in 1934 [19]. Of those, one patient survived up to two years. Hilaris, who was a pioneer in the development of ^125^I seeds for implantation for the treatment of pancreatic carcinoma, published a study of 98 patients receiving seed implants that responded with a median survival of 7 months [20], with 1 patient surviving for five years. Pain control was achieved in 65% of patients and lasted between 5 and 47 months (with a median of 6 months).

In a review study by Morrow et al., no difference in survival between patients treated with interstitial brachytherapy and patients treated by surgical resection at the same institution were observed [21]. The median survival time was 7 months, and at least one patient survived up to five years. Pain control was achieved in 65% of the patients [22]. Syed et al. reported 18 patients treated with biliary bypass surgery, ^125^I interstitial brachytherapy, and EBRT [23]. Ten patients with the interstitial brachytherapy were "sandwiched" between two courses of EBRT. Typically, patients received 30 Gy EBRT following biopsy and bypass surgery, then 2 weeks later an additional interstitial brachytherapy of 100–150 Gy, and then an additional 15–20 Gy EBRT was administered 3–4 weeks after interstitial implantation. The results showed a 13 month median survival time in 12 patients with head and body pancreatic carcinoma. ^125^I seed implantation has been attempted in patients with locally advanced pancreatic carcinoma, and no difference in overall survival was found compared with the use of other techniques [24,25].

In this study, the interstitial needle position and distribution were determined using ultrasound supervision and with the intent to spare at least 1 cm from nearby or normal tissues including the internal pancreatic duct and small blood vessels. The placement of an omental fat pad over the implanted volume was also used to protect the gastric and transverse colon mucosa from irradiation. Our results indicate that the local control of disease was achieved in 78.6% of all patients. 87.5% (7/8) of all patients experienced complete and partial pain relief and shown satisfactory palliative effect. The overall 1-, 2- and 3-year survival rates were 33.9%, 16.9% and 7.8%, respectively with the median survival of 10 months. The survival rate and survival times were found to be the most advantageous for some selected stage II/III patients in this study.

Permanent interstitial administration of radioactive seeds appears to offer consistent and improved local control, although a major drawback is the high rate of perioperative morbidity and mortality. The significant causes of high morbidity of ^125^I seed intraoperative implantation were due to the needles penetrated into pancreatic duct, small blood vessels in the pancreas and/or organ at risk resulting in fistula and abscess formation. The major long-term complication from the combined effects of multimodality treatments has been gastrointestinal bleeding and obstruction [26]. The high incidence of complications maybe related to that the seeds were implanted nearby normal tissues such as gastric, colon and jejunum. The second reason may be the activity of seeds was high. The third reason maybe the doses of seeds beyond the tolerance of normal pancreas tissue. In earlier studies, perioperative mortality was 16% – 25% from acute pancreatitis, fistulization, and abscess formation [23]. Side effects reported in the Hilaris et al., study included 1 patient developing a post-operative mortality, another patient suffered from a pancreatic fistula, 4 patients developed biliary fistula, 4 developed abscesses, 4 developed gastrointestinal bleeding, 6 developed obstruction of the gastrointestinal tract, 5 patients developed sepsis, and 4 patients developed deep venous thrombophlebitis [20]. In comparison, the study by Syed et al. included 8 patients with a poorer prognosis, 2 patients with prolonged wound drainage, 3 patients developed insulin-dependent diabetes, and 2 patients developed other interstitial complications [23]. For this study, perioperative mortality was considerably less than that observed in earlier studies, one patient suffered from chylous fistula, one patient suffered from pancreatitis and one suffered from gastritis, seven patients suffered from low fever, there were no grade III and grade IV toxicity and complications, and less than most series of surgically-treated pancreatic cancer patients published in the literature [22,27].

In conclusion, ^125^I seed implantation with intraoperative ultrasound guidance provides a satisfactory distribution of seeds in tumor mass, minimizes radiation to surrounding organs due to the sharp dose fall-off outside the implanted volume, and generates no damage. We hypothesize that a further improvement in median survival of patients with unresectable pancreatic carcinoma may be obtained with the combined aggressive use of EBRT, systemic chemotherapy.

## Abbreviations

^125^I: iodine-125; LDR: low-dose rate; HDR: high-dose rate; SLD: sublethal damage; TPS: treatment planning system; EBRT: external beam radiotherapy; GTV: gross tumor volume; PTV: planning tumor volume; SRT: stereotactic radiotherapy; CR: complete response; PR: partial response; NS: no response; PD: progressive disease; NRS: numerical rating scale; KPS: karrnofsky performance status.

## Competing interests

The authors declare that they have no competing interests.

## Authors' contributions

JJW conceived of this study, designed, coordinated the study and drafted the manuscript, YLJ, JNL and SQT helped with the data collection, statistical analysis. WQR and DRX carried out the operation. All authors give final approval for the paper to be submitted for publication.

## Supplementary Material

Additional file 1 **Table S1**. Characteristics of ^125^I seed implantation and outcome (n = 14). Click here for file
