# Supplementary material for: Intraoperative ultrasound-guided iodine-125 seed implantation for unresectable pancreatic carcinoma
Source: J Exp Clin Cancer Res. 2009 Jun 23;28(1):88. doi: 10.1186/1756-9966-28-88 (PMC2715376; doi:10.1186/1756-9966-28-88)
Supplement: Additional file 1 — Table S1. Characteristics of 125I seed implantation and outcome (n = 14). [file 1756-9966-28-88-S1.doc]

**Table S1. Characteristics of ^125^I seed implantation and outcome (n=14)**

| No. | Size  (cm) | Number of  ^125^I seeds | Specific activity of per ^125^I seed  (mCi) | D_90_（Gy） | Follow-up time  (m) | NRS of pre-seed implant | NRS of post-seed implant | LP  (m) | Tumor  response | Survival | Cause of death | | | | Survival time  (m) |
| --- | --- | --- | --- | --- | --- | --- | --- | --- | --- | --- | --- | --- | --- | --- | --- |
|  |  |  |  |  |  |  |  |  |  |  | local recurrence | metastasis | metastasis and recurrence | heart disease |  |
| 1 | 4.5x3.0x3.0 | 19 | 0.4 | 80 | 8 | Severe | Mild | 3 | PD | No | Yes | No | No | No | 8 |
| 2 | 3.0×3.0x3.0 | 23 | 0.4 | 120 | 28 | No | No | 28 | CR | No | No | Yes | No | No | 28 |
| 3 | 3.9x2.8x2.8 | 24 | 0.4 | 110 | 24 | Moderate | No | 24 | CR | No | No | Yes | No | No | 24 |
| 4 | 3.0×4.0×4.0 | 28 | 0.5 | 115 | 9 | Severe | No | 6 | PR | No | No | No | Yes | No | 9 |
| 5 | 5.0×5.0×4.5 | 34 | 0.6 | 115 | 5 | Severe | Mild | 5 | PR | No | No | No | No | Yes | 5 |
| 6 | 3.0×3.0x3.0 | 10 | 0.5 | 60 | 3 | No | No | 3 | PR | No | No | Yes | No | No | 3 |
| 7 | 4.1×4.0x4.0 | 28 | 0.5 | 80 | 9 | No | No | 3 | PD | No | No | No | Yes | No | 9 |
| 8 | 2.5×3.0×3.3 | 20 | 0.5 | 125 | 8 | No | No | 3 | PD | No | Yes | No | No | No | 8 |
| 9 | 4.0×4.0x4.0 | 38 | 0.5 | 120 | 10 | No | No | 10 | PR | No | No | Yes | No | No | 10 |
| 10 | 5.0×8.0×5.0 | 75 | 0.5 | 120 | 14 | No | No | 14 | CR | No | No | Yes | No | No | 14 |
| 11 | 4.0×4.0×4.0 | 49 | 0.5 | 130 | 14 | Moderate | Mild | 14 | PR | No | No | Yes | No | No | 14 |
| 12 | 5.0×4.0×4.0 | 58 | 0.5 | 140 | 11 | Severe | No | 11 | PR | No | No | Yes | No | No | 11 |
| 13 | 3.0×4.0×5.0 | 73 | 0.5 | 140 | 43 | Moderate | No | 43 | PR | Yes | - | - | - | - | 43 |
| 14 | 6.0×5.0×4.0 | 44 | 0.6 | 120 | 5 | Severe | Moderate | 4 | PR | Yes | - | - | - | - | 5 |

Abbreviations: *RR*: response rate; *m*: months; *LP: local progression*; *CR*: complete response; *PR*: partial response; *PD*: progressive disease
